# Service Evaluation of Transition Pathway and Audit of National Institute of Clinical Excellence (NICE) Guidelines Compliance

**DOI:** 10.1192/bjo.2025.10495

**Published:** 2025-06-20

**Authors:** Umama Khan, Basanta Sapkota, Parvathy Suresh, Amaia Del-Pozo-Ugalde, Jeremy Turk

**Affiliations:** 1 Hampshire and Isle of Wight NHS Foundation Trust, Isle of Wight, United Kingdom

## Abstract

**Aims:** This project aimed to evaluate our transition service for young people, from Child & Adolescent Service (CAMHS) to Adult Mental Health Services (AMHS), and to audit NICE transition guidelines compliance.

**Methods:** A retrospective case note survey of complex patients who had transitioned between January 2021 and September 2024 was undertaken. NICE Guideline standards on transition were compared with current practices.

**Results:** All individuals had been seen by a consultant psychiatrist prior to transitioning, usually with diagnoses confirmed, and medications stabilized.

37 participants were female and 7 male. 38 were transferred to community mental health team (CMHT), 3 to a learning disability team and 3 to early intervention in psychosis service.

13 participants had a diagnosis of bipolar affective disorder. 21 had a diagnosis of autism spectrum disorder (ASD) and 5 had attention deficit hyperactivity disorder (ADHD). A few awaited diagnoses confirmation. Emotionally unstable personality disorder was the second most common diagnosis, seen in 8 cases.

Individuals with severe anorexia nervosa and possible autism proved the most difficult to engage in treatment following transition. Most individuals continued to be managed in the community. Only 3 required brief admission to hospital for a maximum stay of 3 days.

Only one had contact with the criminal justice system.

Two continued to receive care from CAMHS post 18th birthday, as they didn`t meet the adult service eligibility criteria.

We compared our current practices with NICE standards. There was good compliance with most, other than Standard 1, regarding age at transition planning. Adult service policy was to identify a named worker only a month before the young persons` 18th birthday. Hence, most individuals transitioned aged 17 years and 11 months.

There was NICE compliance for having a coordinated transition plan, a named worker to coordinate transition care and support before, during and after transition., and a patient meeting practitioner from each anticipated adult service.

**Conclusion:** This review has helped us in confirming that our transition pathway is largely effective in transitioning complex and enduring cases to adult services and has identified gaps which require attention. We believe that having a dedicated consultant psychiatrist providing continuity of care, pre and post transfer has been pivotal in reaching these goals.

Additionally, good and early patient preparation, and focused, prioritised, multidisciplinary support for complex cases has been crucial.

